# Forecasting daily COVID-19 cases with gradient boosted regression trees and other methods: evidence from U.S. cities

**DOI:** 10.3389/fpubh.2023.1259410

**Published:** 2023-12-11

**Authors:** Anindya Sen, Nathaniel T. Stevens, N. Ken Tran, Rishav R. Agarwal, Qihuang Zhang, Joel A. Dubin

**Affiliations:** ^1^Department of Economics, University of Waterloo, Waterloo, ON, Canada; ^2^Department of Statistics and Actuarial Science, University of Waterloo, Waterloo, ON, Canada; ^3^School of Public Health Sciences, University of Waterloo, Waterloo, ON, Canada; ^4^Cheriton School of Computer Science, University of Waterloo, Waterloo, ON, Canada; ^5^Department of Epidemiology, Biostatistics and Occupational Health, McGill College, Montreal, QC, Canada

**Keywords:** daily COVID-19 cases, epidemiological surveillance, Metropolitan Statistical Areas, Gradient Boosted Regression Trees, Seasonal Autoregressive Integrated Moving Average (SARIMA), Susceptible, Infectious, or Recovered (SIR), Linear Mixed Effects

## Abstract

**Introduction:**

There is a vast literature on the performance of different short-term forecasting models for country specific COVID-19 cases, but much less research with respect to city level cases. This paper employs daily case counts for 25 Metropolitan Statistical Areas (MSAs) in the U.S. to evaluate the efficacy of a variety of statistical forecasting models with respect to 7 and 28-day ahead predictions.

**Methods:**

This study employed Gradient Boosted Regression Trees (GBRT), Linear Mixed Effects (LME), Susceptible, Infectious, or Recovered (SIR), and Seasonal Autoregressive Integrated Moving Average (SARIMA) models to generate daily forecasts of COVID-19 cases from November 2020 to March 2021.

**Results:**

Consistent with other research that have employed Machine Learning (ML) based methods, we find that Median Absolute Percentage Error (MAPE) values for both 7-day ahead and 28-day ahead predictions from GBRTs are lower than corresponding values from SIR, Linear Mixed Effects (LME), and Seasonal Autoregressive Integrated Moving Average (SARIMA) specifications for the majority of MSAs during November-December 2020 and January 2021. GBRT and SARIMA models do not offer high-quality predictions for February 2021. However, SARIMA generated MAPE values for 28-day ahead predictions are slightly lower than corresponding GBRT estimates for March 2021.

**Discussion:**

The results of this research demonstrate that basic ML models can lead to relatively accurate forecasts at the local level, which is important for resource allocation decisions and epidemiological surveillance by policymakers.

## 1 Introduction

On May 5, 2023, the World Health Organization (WHO) officially ended the global COVID-19 emergency, referring to increased population immunity, fewer deaths, and reduced pressure on hospitals. The COVID-19 pandemic, which was first declared an international crisis by WHO on January 30, 2020, resulted in severe lockdowns, closure of international borders, devastating economic costs upheaval and the deaths of at least seven million people across the world.^1^ Hundreds of published and working research papers have attempted to evaluate the efficacy of traditional statistical models and Machine Learning (ML)/Artificial Intelligence (AI) methods in forecasting COVID-19 cases. Accurate short-term forecasts can inform government decision-making in terms of resource allocation to health practitioners and hospitals, as well as in deciding on the magnitude and severity of lockdowns and timing of re-openings.

The focus on ML and AI methods might be explained by the poor performance of Susceptible-Infected-Removed (SIR) models—traditionally used by epidemiologists to predict the spread of infectious diseases—in forecasting daily COVID-19 counts (1–3). However, many sophisticated statistical methods developed for COVID-19 modeling and forecasting, such as the models from the Institute of Health Metrics and Evaluations, the University of Texas at Austin, and the Los Alamos National Laboratory, have also yielded unsatisfactory results (4). Hence, there is value in identifying alternative models that are relatively easy to implement and interpret, and that are capable of producing accurate predictions. This study evaluates the efficacy of Gradient Boosted Regression Tree (GBRT), Susceptible, Infectious, or Recovered (SIR), Seasonal Autoregressive Integrated Moving Average (SARIMA), and Linear Mixed Effects (LME) models in forecasting daily trends in COVID-19 cases across 25 cities in the U.S. (Albuquerque, Atlanta, Baltimore, Boston, Charlotte, Chicago, Cleveland, Dallas, Denver, Detroit, Houston, Indianapolis, Los Angeles, Louisville, Memphis, Miami, New York, Oklahoma, Phoenix, Pittsburgh, Portland, Sacramento, San Francisco, Seattle, Tampa). We forecast daily COVID-19 case rates one-week and four-weeks ahead over different testing periods. The models chosen are reasonably basic, but this choice is intentional and motivated by the desire to explore the efficacy of simpler models that are relatively easily interpretable and computationally efficient, while also crossing disciplinary boundaries to benchmark the performance of traditional methods employed by researchers across different fields.

The choice of these forecasting models and periods is consistent with other studies. For example, Chumachenko et al. (5) used Random Forest, K-Nearest Neighbors, and Gradient Boosting methods to forecast COVID-19 cases for Germany, Japan, South Korea, and Ukraine with respect to 3, 7, 10, 14, 21, and 30 days. The objective of the study is similar to ours, in terms of assisting public health agencies to identify models that could generate predictions to address various pandemic containment challenges. Krivtsov et al. (6) is a relatively recent example of research based on more complex Neural Network (NN) methods using data for the same countries. Mohammadi and Chumachenko (7) employ ARIMA methods to forecast cases for Ukraine. Other examples of ARIMA based studies at the country level include Dansana et al. (8), Singh et al. (9), and Sahai et al. (10). Devaraj et al. (11) use ARIMA and other deep learning methods. Fang et al. (12) compare the performances for XGBoost and ARIMA specifications using U.S. time-series data and Liu et al. (13) employ ARIMA models to study US national data as well.

Our focus on short-term predictions is guided by the importance of such forecasts for the allocation of local health resources, such as the supply of personal protective equipment (PPE), adequate testing infrastructure, and the availability of hospital care teams. Further, our use of data across several U.S. Metropolitan Statistical Areas (MSAs) or cities is a contribution to the literature as most studies rely on either cross-country, national, state/province, or county level data. While employing aggregated data has benefits, identifying models that are capable of relatively accurate forecasts at the local level can result in more targeted decisions by policymakers. In this respect, we are unaware of any study that has attempted to forecast COVID-19 case counts using a panel of MSAs in the United States. Our study is also a contribution to the literature given that a large fraction of studies, which have attempted to forecast the spread of COVID-19 in the U.S., have not provided any benchmarking of their forecasts against the truth, or stated their limitations (14). Constructing forecasting models at a local level is challenging, given the need to account for unobserved jurisdiction level heterogeneity, and corresponding volatility in daily COVID-19 cases which we observe for several U.S. cities. Such volatility often disappears when data are aggregated across jurisdictions, resulting in a smoother time-series of observations in training datasets, and hence, more accurate predictions. However, such predictions may not be very useful for policymakers interested in epidemic trends within a specific jurisdiction.

A primary objective of our study is to evaluate the performance of traditional SIR models employed by epidemiologists, given findings on the inaccuracy of COVID-19 case forecasts, relative to other methods. A possible explanation behind the poor performance of SIR models is because of the need for properly accounting for relevant geographical characteristics such as the number and distribution of outbreaks, and population size and density (15). The results from SIR modeling are compared to GBRT models intended to evaluate the efficacy of a common ML model. Besides being used by other research, our choice of GBRTs is also motivated by the ease in which models can be implemented by policymakers with limited knowledge of Machine Learning or Artificial Intelligence methods. SARIMA modeling is commonly used for time-series forecasting by economists and is especially useful when the modeled data has pronounced trend and seasonality. LME models are popular with researchers working at the intersection of statistics and health. They enable the researcher to flexibly control for the potentially confounding effects of unit-specific (in this paper, city-specific) heterogeneity through the accommodation of random effects, which themselves accommodate the correlation of within-city repeated measures over time. LME specifications also allow information to be borrowed across geographies, which might result in more accurate predictions, especially for cities that experience considerable within-city variation during the training phase and whose case rates may be spatially correlated with other cities. We do not explore the performance of Neural Network or Long Short-Term Memory (LSTM) models as we restrict our analysis of methods that are relatively straightforward to implement. Finally, given results from other studies [for example, (16–20)], which suggest benefits from the use of ML/statistical models in tandem with social mobility/internet data, we downloaded Apple mobility data for each MSA to evaluate their potential in generating more accurate forecasts.

Citing all papers that have employed such methods in forecasting daily COVID-19 case trends is beyond the scope of this study. Focusing on studies that have employed either county or state/province level data, Altieri et al. (21) and Liu et al. (22) construct one- and two-week-ahead forecasts (of case or death counts) using either different types of linear and exponential predictors or Bayesian methods. Chu and Qureshi (1), Chen et al. (2), Stevens et al. (23), and Sen et al. (24) find different autoregressive time series and ML models to be capable of comparable or superior short-term out-of-sample forecasts of daily cases relative to SIR models. In terms of cities, Wathore et al. (25) rely on deep learning models such as LSTM to forecast cases for 8 cities in India, U.S., and Sweden. Their study contains a summary of other LSTM based papers. Devaraj et al. (11) also contains a detailed discussion of deep learning-based papers. Zhang et al. (26) develop a hybrid predictive model of COVID-19 cases based on autoregressive and LSTM models, which they test for 8 counties in California and some countries.

The limited amount of research exploiting variation across cities over time is probably because of the lack of publicly available data. For U.S. cities, we surmount this difficulty by employing county level data collected by the New York Times and constructing corresponding MSA level daily case counts. As a result, we are able to clearly see patterns and differences in COVID-19 cases across some major U.S. metropolitan areas during the first and second waves of the pandemic. Another contribution of our study is that we produce forecasts during time periods that coincide with the peak of the second wave of infections, specifically during November 15 - December 12, 2020, along with subsequent time periods, which saw significant declines in cases counts for many MSAs. This is in contrast to many early published studies that are focused on forecasts of the first wave and Summer of 2020.

Our choice of this time period is also motivated by the considerable volatility in daily case counts observed across cities during November–December 2020, making the forecasting exercise more challenging. We also conduct forecasting exercises for January–March 2021 as well, to evaluate model efficacy during periods of significant declines in daily case counts. The need to ensure that training data for forecasting models capture dynamic changes in the spread of the virus has been noted by other studies (11, 25, 27). Employing data from these time periods is further justified given the rise in population vaccination rates from March 2021 onwards, and the widespread use of home testing kits from late 2021 onwards, which impacts the reliability of official statistics, given the possibility of under-reporting of positive tests to health authorities.

For most MSAs, GBRT and SARIMA models produce forecasts for November 15–December 12, 2020, with lower Median Absolute Percentage Errors (MAPEs) than corresponding one-week ahead predictions produced by LME and SIR models and are consistent with other studies that find SIR models to produce inaccurate forecasts of the incidence and spread of COVID-19. Apple mobility data do not make a significant difference for the forecast accuracy of SARIMA models. With respect to 7-day ahead forecasts, GBRTs produce MAPEs lower than SARIMA models for most MSAs for the November-December 2020, January, and February 2021 testing periods. On the other hand, SARIMA MAPEs are lower for the March 2021 testing period. Likewise, the 28-day ahead forecasts produced by SARIMA models generate lower MAPE values in March 2021. However, for the other months considered, 28-day ahead GBRT forecasts tend to be associated with lower MAPE values.

## 2 Methods and materials

### 2.1 Data

Daily COVID-19 case data at the county level were downloaded from the Github repository maintained by the New York Times^2^ We note that the COVID-19 data maintained by the New York Times and John Hopkins University^3^ have been widely employed by researchers. Using Federal Information Processing System (FIPS) codes, county-level data were mapped to Metropolitan Statistical Areas (MSAs), which include city cores and adjoining suburbs, to provide daily case counts at the MSA level. We herein use the terms city and MSA interchangeably. Our choice of MSAs was based on investigating COVID-19 trends in the largest cities across the U.S, while ensuring representation across different regions.

**Figure 1:**
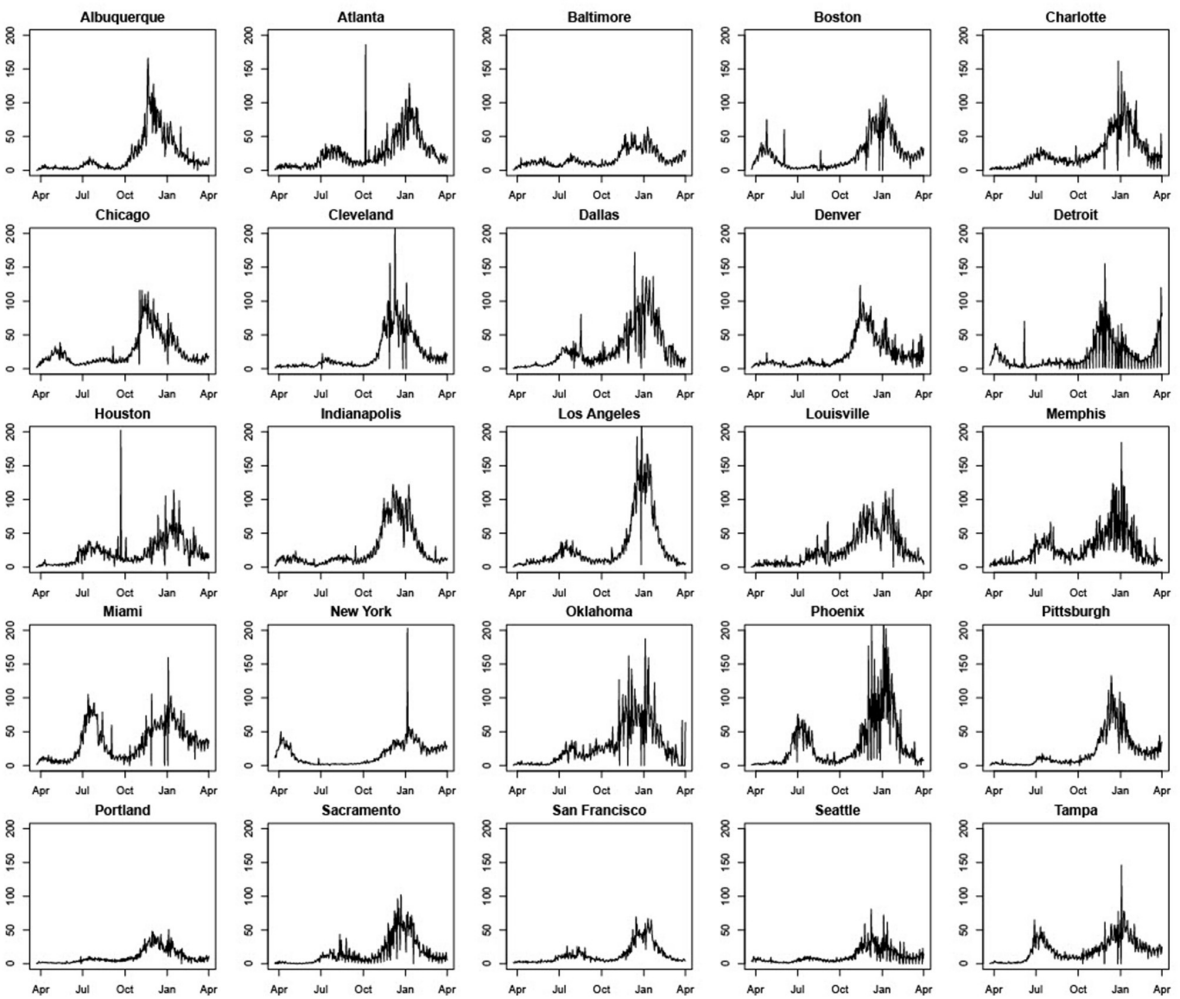
Standardized daily case counts per 100,000 people in 25 U.S. cities (March 22, 2020–March 31, 2021).

Figure 1 shows significant variation in COVID-19 daily cases across MSAs and over time. Some MSAs such as Miami, Phoenix, Oklahoma City, Atlanta, Dallas, Charlotte, Tampa, Houston, San Francisco, and Sacramento had much higher daily case counts per 100,000 of population during July and August of 2020, relative to the first wave in March and April of the same year. In contrast, New York, Boston, and Chicago had much higher case counts per 100,000 of population or per capita daily cases during the first wave. Most cities experienced a decline in COVID-19 cases during September, which was succeeded by a rapid increase during November and December that coincided with intensive campaigning associated with the U.S. Presidential Election. Increases in daily case counts during this time period were succeeded by declines that began sometime during December 2020 or January 2021 for almost all MSAs, and that continued through March 2021.

Five other points should be noted. First, some cities such as Atlanta, Boston, Cleveland, and Houston, have peaks in daily case counts that are clearly anomalous with previous and successive trends. This can be traced to errors in data collection and reporting. The most common explanation we were able to find is that spikes are attributable to the reporting of a significant backlog of cases that, for some reason, were not reported correctly earlier.^4^ Second, most cities seem to experience considerable volatility in daily COVID-19 case counts during November and December 2020. Third, days with zero cases reported are likely due to batch reporting in previous days or non-working days and were changed to 0.5 to prevent our performance metrics from having nonsensical results when dividing by zero, as well as to allow us to model the natural logarithm of the counts (specifically, the log of standardized case counts per 100,000) for the LME and SARIMA models. Fourth, unlike other MSAs, Detroit experienced a significant rise in daily case counts during March 2021. Daily COVID-19 cases also rose modestly during March 2021 in Pittsburgh and Boston. Fifth, across all MSAs, case rates are clearly non-stationary with pronounced trend and seasonality. Effective modeling and forecasting should account for this correlation structure. Table 1 contains means and standard deviations of standardized daily case counts per 100,000 for each MSA for our training period of March 22, 2020–November 14, 2020.

**Table 1:** Sample statistics.

**Sample mean (s.d.) of daily COVID-19 standardized cases per 100K (March 22–Nov 14, 2020)**				
**Albuquerque**	**Atlanta**	**Baltimore**	**Boston**	**Charlotte**
9.65	14.36	10.98	11.69	13.50
(12.53)	(14.69)	(5.47)	(11.02)	(8.41)
**Chicago**	**Cleveland**	**Dallas**	**Denver**	**Detroit**
18.84	8.59	14.59	13.03	11.74
(19.39)	(9.81)	(11.77)	(16.58)	(12.71)
**Houston**	**Indianapolis**	**Los Angeles**	**Louisville**	**Memphis**
14.30	13.52	12.81	15.17	19.54
(15.95)	(12.82)	(7.97)	(15.02)	(13.49)
**Miami**	**New York**	**Oklahoma**	**Phoenix**	**Pittsburgh**
23.81	8.45	15.42	16.06	6.49
(23.42)	(10.05)	(15.54)	(17.72)	(6.47)
**Portland**	**Sacramento**	**San Francisco**	**Seattle**	**Tampa**
5.52	7.16	7.06	6.23	12.87
(5.06)	(7.18)	(4.85)	(4.54)	(12.18)

### 2.2 Methods

We begin by introducing notation that will be used for all the different methods introduced in this section. Let *t* = 0, 1, 2*, …, T* represent time, which here we take to be measured in days. Thus, for a given analysis of an MSA's daily case counts, we will observe a maximum of *T* + 1 time points. Each city is represented by the index *i* = 1, 2*, …, N*, where *N* = 25 in this paper's analyses. The response variable, denoted by *Y*_*i,t*_, is documented COVID-19 case counts per 100,000 people, standardized by the city population size. In particular,


$$\begin{array}{c} Y_{i,t}=\left(\#\text{new cases in city }\text{i }\text{on day }\text{t}/\text{Population size of city i}\right)\\ \times \;100,000 \end{array}$$

represents the per capita daily case count (per 100,000 people) in city *i* at time point *t*. Note that this standardization is necessary to ensure that case rates are comparable across cities of different sizes. For models where lagged case values are included as predictor variables, the earliest first day used for the response will be constrained by the number of lagged terms; for example, if we include predictors *Y*_*t*−1_ and *Y*_*t*−2_, then the first response in the model will be at *t* = 2. In our models specified in the following subsections, the first date to be used for *Y*_*t*_ is March 22, 2020, since this is the date that we start to see (at least some) non-zero case counts in all 25 cities.

As discussed in Section 1, some studies have used social media or cellular data to model population mobility and forecast the incidence and spread of COVID-19. Therefore, we use three separate social mobility indices released by Apple, that measure walking, driving, and transit use, respectively, for certain geographies. While most studies have relied on Google mobility data, Google does not offer publicly available data at the MSA level. The Apple data are collected from Apple Maps app usage through individual iPhones. The indices show changes in relative volume of directions requests per country/region, sub-region, or city compared to a baseline volume on January 13th, 2020. While we do not claim these indices necessarily capture population-wide mobility trends, using these data at least allows us to evaluate the effects of social mobility on daily COVID-19 cases counts, and assess the usefulness of this information in constructing daily predictions of COVID-19 cases in MSAs. Moreover, the walking mobility indicator is highly correlated with temperature and therefore serves as a useful proxy for weather, which is known to be an important factor in transmission (28).

In terms of quantifying the forecasting performance of different models, we consider the Median Absolute Error (MAE) and Median Absolute Percentage Error (MAPE) of predicted daily case counts for each Metropolitan Statistical Area. The MAE is the median of the absolute value of the difference between actual and predicted daily case counts over the chosen testing period, while the MAPE is the median of the absolute value of the corresponding percentage difference. While MAEs are reported, our comparisons are primarily based on the MAPE as it is a standardized metric interpreted equivalently across all cities. Hence, for a city *i*, if the actual observed daily case count is *Y*_*t*_ and the predicted value from a chosen model is Ŷ_*t*_, the MAE is the median absolute value of their difference or |*Y*_*t*_ − Ŷ_*t*_|, calculated from daily values over the testing period. The MAPE is therefore the median of $|\frac{Y_{t}-{Ŷ}_{t}}{Y_{t}}|\times 100$ over the testing sample. For further sensitivity analysis, we also report the proportion of daily forecasts with an absolute percentage forecast error <20%.

#### 2.2.1 Linear Mixed Effects (LME) models

To assess the statistical importance of Apple mobility indicators, we ran LME regressions using *Y*_*i,t*_ (i.e., standardized case count per 100,000 for city *i* at time *j*) as the dependent variable and based on data pooled across cities and over days for the training period. As a sensitivity analysis, we also ran the LME regressions for the time period March 22 - August 31, 2020, before the beginning of the steady rise in case counts for most cities. The LME models considered contain the seven-day lag of the walking index, one and two day lags of the dependent variable along with a weekend indicator variable.^5^ Estimation results are reported in Table 2. First, while these specific results are not reported, we note that lagged dependent variables of a higher order than 3 days were statistically insignificant in these models. Second, one and two-day lags in the dependent variable are statistically significant at the 1% level. Third, the seven-day lagged Apple mobility Walking Index is also statistically significant at the 1% level. These results offer some support to the inclusion of a mobility index in the models used to forecast daily case counts. On the other hand, we note that the inclusion of the Apple mobility index does not significantly improve the fit of the LME models as measured through the AIC and Log-Likelihood, and so its inclusion may not drastically improve a model's forecasting capability.

**Table 2:** Baseline linear mixed effect model summaries (standard errors in parentheses).

	**Mar 22–Aug 31, 2020**	**Mar 22–Nov 14, 2020**	**Mar 22–Aug 31, 2020 with Apple Mobility**	**Mar 22–Nov 14, 2020 with Apple Mobility**
**Estimation results**				
Log cases_*t*−1_	0.49 (0.018)	0.444 (0.012)	0.489 (0.019)	0.435 (0.013)
Log cases_*t*−2_	0.38 (0.015)	0.407 (0.011)	0.371 (0.015)	0.401 (0.012)
Seven day lag Apple Walking Mobility index			0.001 (0.0003)	0.002 (0.0002)
Weekend dummy	Yes	Yes	Yes	Yes
AIC	7,137.503	13,607.74	7,136.557	13,582.3
Log-likelihood	−3,560.75	−6,795.87	−3,559.28	−6,782.16

In terms of the specific LME model:


(1) $$\begin{array}{c} \log\left(Y_{i,t}\right)=\beta_{0}+b_{0,\text{i}}+\beta_{1}\log\left({Y_{i,t-}}_{1}\right)+\beta_{2}\log\left({Y_{i,t-}}_{2}\right)+\beta_{3}WE_{t}\\ +\varepsilon_{i,t}, \end{array}$$

where:

- β_0_ is the population-level intercept;
- *b*_0,i_ is a city-specific random intercept, with *b*_0,i_ ~ N(0^2^, σ_*b*0_);
- β_1_ is the fixed-effect parameter connected with one-day lagged (log) count for city *i*;
- β_2_ is the fixed-effect parameter connected with two-day lagged (log) count for city *i*;
- β_3_ is the fixed-effect parameter connected with the weekend indicator, common to all cities. Specifically, if day t falls on a weekend, then *WE*_t_ = 1, otherwise *WE*_t_ = 0;
- ε_*i,t*_ is the model error term, with **ε**_*i*_ = (ε_*i*,1_*, . . .*, ε_*i,T*_)^*t*^ ~ N_*T*_ (**0**, σ^2^**I**_*T*_), and **ε**_*i*_ independent of *b*_0*, i*_.

Note we have initially assumed that the model error terms are conditionally independent, with the random intercept being the only term to induce correlation between the repeated measures of daily counts within the same city. We also see that all cities have a common *T* repeated measures observed. We found that a modified version of (1) provides a better fit to the standardized city-level daily COVID-19 case count data. Specifically, allowing for heterogeneity in both time-varying predictors, i.e., attaching city-specific random effects to each, and properly handling existing heteroskedasticity among the model error terms was also required, leading to:


(2) $$\begin{array}{c} \log\left(Y_{i,t}\right)=\beta_{0}+b_{0,\text{i}}+\left(\beta_{1}+b_{1,\text{i}}\right)\log\left({Y_{i,t-}}_{1}\right)\\ +\left(\beta_{2}+b_{2,\text{i}}\right)\log\left({Y_{i,t-}}_{2}\right)+\beta_{3}WE_{t}+\varepsilon_{i,t}, \end{array}$$

where the changes relative to (1) are:

- *b*_1,i_ is a city-specific random effect connected to the first-order lagged (log) counts, with *b*_1,i_ ~ N(0^2^, σ_*b*1_);
- *b*_2,i_ is a city-specific random effect connected to the second-order lagged (log) counts, with *b*_2,i_ ~ N(0^2^, σ_*b*2_); we allow for all three random effects to be correlated, though each are independent of **ε**_*i*_;
- **ε**_*i*_ ~ N_*T*_ (**0**, **Σ**), with **Σ** diagonal, but weighted in such a way to remove the original heteroskedasticity in the model error terms.

From (2), we can forecast one time point ahead (e.g., one day ahead), then use that forecast as the first-order lagged term to forecast one additional day ahead (where the prior first-order lagged term is now the second-order lagged term), and so on, until we have forecasted forward the desired number of days. Note that linear mixed effect models have units borrow strength from other units for a given unit's trajectory prediction. Though not all cities are aligned in time in terms of model dynamics, not nearly as much borrowing of strength from other city's predictions will affect a given city's predictions, due to the extensive collection of repeated measures per city paired with the relatively reasonable within-city variation. Based on the results in Table 2, the Apple Walking Index is employed as a predictor for some specifications. Estimation of LME models and forecasts were conducted using the *lme4* package from the R programming language.

#### 2.2.2 Susceptible-Infected-Removed (SIR) model

SIR models are the dominant methodology to model the spread of epidemics; see, for example, Tolles and Luong (29) for further details. The SIR model uses differential equations to describe the dynamic status of an individual switching between three compartments in a population at time *t*: susceptible (*S*_t_), infected (*I*_t_), and removed (*R*_t_) (including recovered and deceased individuals) and is a standard approach employed by epidemiologists to forecast disease spread. *N*_t_ is the total population at time *t* and is identified by *N*_t_ = *S*_t_ +*I*_t_ +*R*_t_, where β is the average number of contacts per infectious person per time unit, γ is the transition rate from *I*_t_ to *R*_t_, and *R*_t_ includes recovered and deceased individuals. The SIR model is then given by the following ordinary differential equations:


(3) $$\begin{array}{c} \frac{\partial S_{t}}{\partial t}=-\frac{\beta I_{t}S_{t}}{N} \end{array}$$

(4) $$\begin{array}{c} \frac{\partial I_{t}}{\partial t}=\frac{\beta I_{t}S_{t}}{N}-\gamma I_{t} \end{array}$$

(5) $$\begin{array}{c} \frac{\partial R_{t}}{\partial t}=\gamma I_{t} \end{array}$$

where β is the average number of contacts per infectious person per time unit and γ is the transition rate from *I*_*t*_ to *R*_*t*_. While the SIR model is based on the modeling of *S*_*t*_, *I*_*t*_, and *R*_*t*_, our focus here is the daily infection numbers and aims to conduct predictions such that the prediction errors are minimized. With the daily number of confirmed cases on day *t* considered as the difference of *S*_*t*_ and *S*_*t*−1_, we calculate the predicted number of daily confirmed cases on day *t*, denoted Ŷ_*t*_(β, γ), as follows:


(6) $$\begin{array}{c} {Ŷ}_{t}\left(\beta ,\gamma \right)=S_{t-1}-S_{t}=I_{t}-I_{t-1}+R_{t}-R_{t-1} \end{array}$$

for *t* = 1, …, *T*, where *T* represents the end of the study period. We implicitly assume that *N*, β, and γ are constant over time.

Then, the parameters β and γ can be obtained by minimizing the squared prediction error


(7) $$\begin{array}{c} PE(\beta ,\gamma )=\sum_{t=1}^{T}{\left\{Y_{t}-{\hat{Y}}_{t}(\beta ,\gamma )\right\}}^{2} \end{array}$$

for β and γ. *Y*_*t*_ is the total number of daily cases in a province at time *t* and is not in per capita terms. We convert the forecasts into per capita terms. Our forecasting procedures model predictions of total daily cases as these are the numbers that are reported and are of interest to policymakers in tracking the incidence and spread of COVID-19. This is consistent with the approach employed by Chen et al. (2). Estimation of the SIR models and construction of forecasts were done using the R programming language. Specifically, we utilized the *EpiDynamics* and *bbmle* packages to estimate the parameters of the SIR model.

#### 2.2.3 Box-Jenkins time series modeling

In this paper we also employ the Box-Jenkins class of time series models referred to herein as SARIMA (Seasonal Autoregressive Integrated Moving Average) models and we use the notation log(*Y*_*i,t*_) ~ SARIMA(*p, d, q*)(*P, D, Q*)[*m*], where, again, *Y*_*i,t*_ is the standardized case count per 100,000 in city *i* on day *t*. Thus, for each city *i* = 1, 2*, . . ., N*, we separately fit a model of the form


(8) $$\begin{array}{c} \phi \left(B\right)\Phi {\left(B^{m}\right)}^{D}{\left(1-B\right)}^{d}log\left(Y_{i,t}\right)=\theta \left(B\right)\Theta {\left(B^{m}\right)}^{D}\varepsilon_{i,t}, \end{array}$$

where *B* is the backshift operator^6^ and *m* is the period of the seasonal component, which here we take to be 7 given the daily data. We also assume that ε_*i,t*_ $\underset{\sim }{{\;}^{i\;i\;d}}$N(0, σ^2^) and we define ϕ(*z*), θ(*z*), Φ(*z*), and Θ(*z*) respectively to be the following *p*^th^ order, *q*^th^ order, *P* ^th^ order, and

*Q*^th^ order polynomials:


$$\begin{array}{c} \phi \left(z\right)=1{-\phi }_{1}{z-\phi }_{2}z^{2}-...-\;\phi_{p}z^{p}\\ \theta \left(z\right)=1+\theta_{1}z+\theta_{2}z^{2}+...+\;\theta_{p}z^{q}\\ \Phi \left(z\right)=1-\Phi_{1}z-\Phi_{2}z^{2}-...-\;\Phi_{p}z^{P}\\ \Theta \left(z\right)=1+\Theta_{1}z+\Theta_{2}z^{2}+...+\;\Theta_{p}z^{Q} \end{array}$$

Simple algebra shows that the response for city *i* on day *t*, log(*Y*_*i,t*_), is therefore a weighted sum of historical log case counts. Note that different values of the non-seasonal (*p, d, q*) and seasonal (*P, D, Q*) orders give rise to different configurations of the model, accounting for different forms of correlation structure in the observed time series. We choose the values of *p, d, q, P, D, Q* that minimize the corrected Akaike information criterion to ensure the model fits the observed data well. The models themselves are fit using maximum likelihood estimation. As with the LME model, the 7-day lag of the Apple Walking Index is also employed as an exogenous variable in some specifications. These time-series models were estimated employing the *forecast* package in the R programming language.

#### 2.2.4 Gradient Boosted Regression Trees (GBRTs)

Gradient Boosted Regression Trees are a commonly used ML algorithm based on decision trees to produce forecasts of particular outcomes. The algorithm sequentially tests the predictive power of different trees to reduce forecast errors, until no further improvements can be made. These predictions are then combined through a weighted average of regressions to produce a final prediction. Although there are other more sophisticated ML methods that can be employed to generate predictions, our choice of GBRTs was motivated by their relative simplicity and interpretability, as well as their ease of implementation through software such as R and Python. This makes them an attractive choice for policymakers with limited resources and fairly limited experience with machine learning.

We assume again our sample contains *T* + 1 observations for each MSA *i*. In particular, we assume that we observe the response variable *Y*_*i,t*_ (standardized case count per 100,000 for city *i* at time *j*) and a vector of predictors given by **X**_*i,t*_ for *i* = 1,2,…, *N* and *t* = 0, 1, 2, …., *T*. The model that forecasts *Y*_*i,t*_ based on **X**_*i,t*_ is a weighted additive model of the form


(9) $$\begin{array}{c} Y_{i,t}=\sum_{k=1}^{K}\alpha_{k}f_{k}(X_{i,t})+\varepsilon_{i,t}, \end{array}$$

where *f*_*k*_(·) for *k* = 1,2, …, *K* are regression trees, α_*k*_ are weights, and ε_*i,t*_ is an error term. The algorithm estimates both the weights α_*k*_ for *k* = 1,2, …, *K* and *f*_*k*_(·) by sequentially minimizing a penalized differentiable convex loss function of $Y_{t}-\sum_{k=1}^{K}\alpha_{k}f_{k}(X_{k})$ with respect to both α_*k*_ and *f*_*k*_(·) over *K* boosting iterations for *k* = 1,2, …, *K*. These estimates can then be used to produce forecasts of *Y*_*i,t*_. For the **X**_*i,t*_ variables, we employ: the weekend dummy variable; city-specific fixed effects; one- to seven-day lags of the dependent variable; and seven- to ten-day lags of all the Apple mobility variables. GBRTs enable the use of all Apple mobility variables and more lagged dependent variables without concern of collinearity issues, as weak predictor variables are given less weight in constructing forecasts. The *XGBoost* package in Python was used to estimate the GBRT models and construct predictions. Code for all of the above models and algorithms are available upon request.

## 3 Results

**Figure 2:**
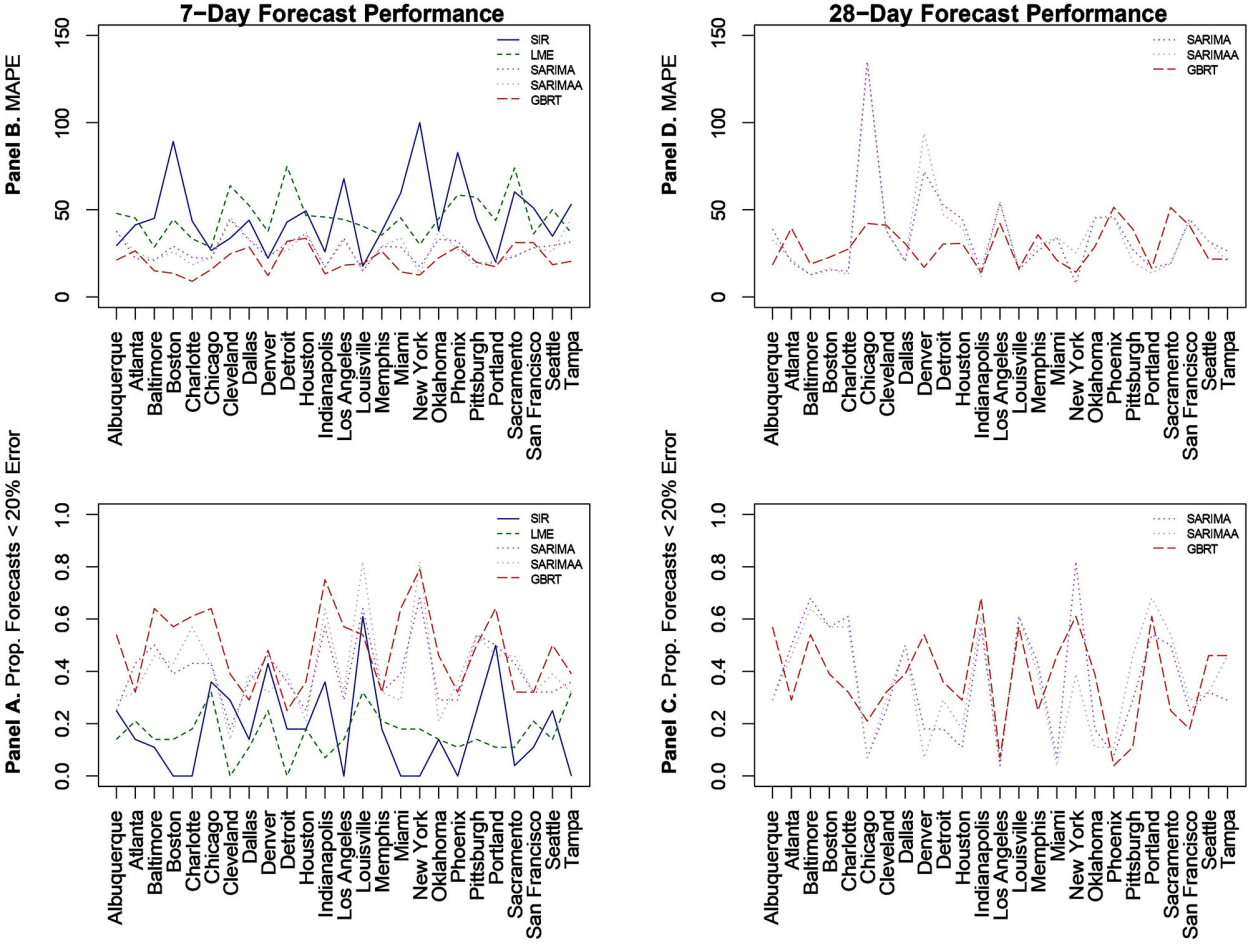
Forecast performance as a function of MSA for 7-day **(A, B)** and 28-day **(C, D)** forecasting strategies for November 15-December 12, 2020. **(B, D)** Quantify forecast performance by MAPE (smaller values are better); **(A, C)** do so using the proportion of daily predictions with a forecast error lower than 20% (larger values are better).

We begin with predictions that are 1 week or 7-day ahead forecasts for each of the 25 MSAs for November 15–December 12, 2020 based on all the models. Given the poor performance of SIR and LME models, we then evaluate the sensitivity of our findings by constructing forecasts from SARIMA and GBRT models for different time periods in January, February, and March of 2021. Figure 2 visualizes MAPE values and the proportion of daily predictions with a forecast error lower than 20% for daily standardized COVID-19 cases (per 100,000 of population) based on LME, SIR, SARIMA, SARIMA models with the Apple Walking Index (referred to as SARIMAA), and GBRTs for each MSA. Note the values displayed in these plots are also tabulated in Table 3. In Figure 2, Panels A and C report the proportion of daily predictions with a forecast error lower than 20% with respect to 7-day and 28-day forecast performances, respectively. Panels B and D contain corresponding visualizations for MAPE values based on 7-day and 28-day forecast errors. For Panels B and D we seek to identify the methodology that has the lowest average MAPE values across cities. On the other hand, for Panels A and C we are interested in the methodology which has the highest average of the proportion of daily predictions with a forecast error lower than 20%, across cities.

**Table 3:** 7-day forecasting performance from LME, SIR, and SARIMA models November 15-December 12, 2020.

**City**	**LME**			**SIR**			**SARIMA**			**SARIMAA**			**GBRT**		
	**MAE**	**MAPE**	**Prop**<**20% FE**	**MAE**	**MAPE**	**Prop**<**20% FE**	**MAE**	**MAPE**	**Prop**<**20% FE**	**MAE**	**MAPE**	**Prop**<**20% FE**	**MAE**	**MAPE**	**Prop**<**20% FE**
Albuquerque	43.97	47.93	0.14	25.91	29.31	0.25	28.12	37.97	0.25	32.79	37.21	0.29	18.4	21.69	0.46
Atlanta	13.89	45.27	0.21	12.72	41.31	0.14	6.59	22.2	0.43	8.99	25.63	0.32	9.79	21.66	0.43
Baltimore	9.3	28.63	0.14	18.71	45.09	0.11	7.08	21.09	0.5	7.1	21.18	0.46	6.18	18.12	0.57
Boston	19.01	44.44	0.14	33.23	89.18	0	12.02	29.27	0.39	9.93	25.78	0.43	6.21	15.72	0.64
Charlotte	14.67	33.43	0.18	19.29	43.59	0	9.51	22.8	0.43	8.85	18.38	0.57	5.66	12.61	0.64
Chicago	22.26	28.47	0.32	20.03	26.61	0.36	13.99	22.01	0.43	14.17	22.47	0.43	11.81	14.45	0.61
Cleveland	41.95	63.77	0	22.28	33.65	0.29	33.32	44.39	0.18	33.38	45.73	0.14	19.85	27.21	0.39
Dallas	27.69	52.43	0.11	20.67	44.03	0.14	17.89	32.81	0.36	17.65	32.84	0.39	17.6	30.49	0.36
Denver	26.57	37.55	0.25	15.43	22.19	0.43	17.13	22.81	0.46	18.29	25.31	0.32	10.76	13.81	0.68
Detroit	43.82	74.78	0	28.71	42.99	0.18	20.33	31.05	0.36	13.64	26.16	0.39	25.04	39.86	0.18
Houston	11.51	46.72	0.18	14.1	49.37	0.18	11.08	36.37	0.25	11.15	37.35	0.21	9.1	33.18	0.36
Indianapolis	38.5	45.82	0.07	25.18	25.84	0.36	15.7	17.32	0.57	14.03	16.85	0.64	6.89	8.77	0.71
Los Angeles	19.88	44.47	0.14	31.99	67.81	0	14.56	33.86	0.29	14.81	32.15	0.32	10.76	22.53	0.39
Louisville	28.24	40.63	0.32	12.21	17.63	0.61	10.03	15.03	0.64	9.77	15.42	0.82	8.95	11.99	0.64
Memphis	15.07	35.55	0.21	18.69	37.78	0.18	14.02	29.11	0.32	14.15	29.03	0.32	15.64	28.53	0.36
Miami	23.62	45.33	0.18	32.43	59.62	0	15.35	28.69	0.39	17.83	33.77	0.29	11.6	22.33	0.43
New York	6.53	30.08	0.18	25.95	99.97	0	4.28	17.56	0.68	3.42	13.41	0.82	3.27	14.04	0.75
Oklahoma	31.79	44.99	0.14	26.61	37.92	0.14	25.81	33.28	0.29	26.26	37.77	0.21	16.72	25.09	0.32
Phoenix	34.4	58.45	0.11	42.71	82.78	0	17.74	32.23	0.29	16.28	27.28	0.36	18.23	32.29	0.32
Pittsburgh	28.53	57.17	0.14	21.81	44.38	0.25	9.83	19.45	0.54	9.86	17.32	0.54	10.4	21.2	0.43
Portland	14.38	43.91	0.11	6.2	20.05	0.5	5.46	19.77	0.5	6.15	21.26	0.46	4.9	15.83	0.57
Sacramento	27.5	73.97	0.11	20.56	60.25	0.04	9.14	23.27	0.43	9.27	23.72	0.46	12.75	27.41	0.32
San Francisco	7.13	36.19	0.21	9.87	51.06	0.11	5.8	28.45	0.32	5.13	28.16	0.32	6.88	5.97	30.58
Seattle	15.39	50.07	0.14	9.89	34.83	0.25	8.44	29.59	0.32	7.4	25.92	0.39	6.22	20.1	0.5
Tampa	12.3	36.56	0.32	15.69	53.29	0	9.59	31.74	0.36	11.48	44.97	0.32	5.63	19.52	0.57

### 3.1 LME and SIR 7-day ahead

With respect to 7-day ahead predictions, LME models produce lower MAPEs for 9 MSAs, relative to SIR models. However, the LME MAPEs are also quite high, with Baltimore having the lowest one at 28.63%. SIR models also do not offer consistently accurate forecasts across cities. With the exception of Denver, Louisville, and Portland, SIR MAPE values are always above 25% for such models. Further, SIR models perform poorly on the basis of the proportion of daily forecasts falling within 20% of the actual value, as there are 7 cities for which the value of this measure is zero. It is also important to note the extremely high MAPE values of approximately 90% and 100% for Boston and New York, respectively, as these two cities experienced extremely high per capita daily case counts during the first wave of infections. Further, the MAPE results indicate that even the use of a long training period is not sufficient to enable the SIR models to acknowledge the subsequent downward trend in daily infections and readjust to generate more accurate forecasts for the testing period.

### 3.2 SARIMA 7-day ahead

Relative to SIR and LME forecasts, SARIMA models produce forecasts with MAPEs that are lower for the majority of cities. SARIMA forecasts yield MAPEs smaller than 20% for four cities and smaller than 25% for six more cities. Another observation is that SARIMA modeling generates predictions that are within tighter bounds for almost each city. Specifically, there is only one MSA (Cleveland) with a MAPE >40% with SARIMA modeling. On the other hand, the number of MSAs with MAPEs >40% based on LME and SIR models are 17 and 15, respectively. Another measure that supports the notion that SARIMA models produce superior forecasts to SIR and LME methods, is the fact that no MSA has a proportion of absolute forecast errors lower than 20% equal to zero, based on SARIMA predictions. Further, for 11 MSAs the proportion of such observations, generated from SARIMA models, is >0.4. Based on these findings, we conclude that SARIMA is a superior forecasting strategy for the vast majority of MSAs. Employing the Apple Walking Index does not make much of a difference in SARIMA forecasts. As we can see in Figure 2, with the exception of a few MSAs, SARIMA MAPEs with and without the Apple Walking Index are comparable. For seventeen cities the difference between MAPEs is <3 percentage points. The proportion of daily forecasts falling within 20% of the actual values are also quite comparable between SARIMA forecasts with and without Apple mobility data.

### 3.3 GBRT 7-day ahead

For most MSAs, GBRTs produce more accurate forecasts of the methods considered. Specifically, GBRTs generate predictions with lower MAPEs for 17 MSAs, relative to corresponding forecasts from SARIMA models with and without the Apple Mobility Index. Eleven MSAs have GBRT forecasts with MAPEs lower than 20%, with Baltimore, Boston, Charlotte, Chicago, Denver, Indianapolis, Miami, and New York having the lowest values. These results suggest that GBRTs are capable of producing relatively accurate 7-day ahead forecasts even during time periods of steep increases and some volatility in daily case counts. The GBRT results are driven by the lagged dependent variables, and not the Apple mobility variables. Specifically, the feature importance scores of the one- to five-day lagged dependent variables range from 0.01 to 0.29. Among the Apple mobility indicators, only the 8-day lagged Driving Index has a feature score above zero, but which is still low at 0.01. These results are consistent with the satisfactory performance of SARIMA specifications without the Apple Walking Index for some MSAs, as the predictors in these models are lagged dependent variables.

### 3.4 SARIMA, SARIMAA, and GBRT 28-day ahead

Based on the noticeably poorer performance of the LME and SIR models, the 28-day ahead forecasts (Figures 2C, D) are based on SARIMA, SARIMAA, and GBRT models only. The predictions are constructed from a single sequence of 28-day ahead forecasts (without model updating) for each of the 25 MSAs for November 15–December 12, 2020. Note the values displayed in these plots are also tabulated in Table 4. Here, we note that SARIMA models produce lower MAPEs for a majority of MSAs, as there are 14 MSAs for which SARIMA MAPEs are lower than corresponding GBRT values. However, when the SARIMA MAPEs are larger than the GBRT MAPEs, they are often *much* larger, and when SARIMA MAPEs are smaller than GBRT MAPEs, they tend only to be *marginally* smaller. Consequently, across all MSAs the average SARIMA MAPE is *higher* than the corresponding GBRT value, despite individual SARIMA MAPEs being smaller for a larger number of cities. As such, GBRTs appear to be the best choice for forecasting both 7-day ahead and 28-day ahead daily standardized COVID-19 case counts for November 15–December 12, 2020.

**Table 4:** 28-day forecasting performance from GBRT and SARIMA models with and without Apple Mobility November 15- December 12, 2020.

**City**	**GBRT**			**SARIMAA**			**SARIMA**		
	**MAE**	**MAPE**	**Prop**<**20% FE**	**MAE**	**MAPE**	**Prop**<**20% FE**	**MAE**	**MAPE**	**Prop**<**20% FE**
Albuquerque	17.01	18.41	0.57	31.17	32.77	0.29	34.61	38.91	0.29
Atlanta	11.9	39.49	0.29	6.59	21.61	0.46	6.46	20.07	0.5
Baltimore	7.48	18.86	0.54	4.34	12.79	0.64	4.33	12.79	0.68
Boston	9.39	22.77	0.39	8.04	16.56	0.57	7.19	15.56	0.57
Charlotte	11.22	27.48	0.32	5.07	12.84	0.57	5.62	14.86	0.61
Chicago	30.41	42.21	0.21	88.57	134.28	0.07	88.29	135.05	0.07
Cleveland	29.51	41.2	0.32	32.69	38.41	0.29	32.45	38.17	0.25
Dallas	19.71	30.59	0.39	11.22	20.43	0.5	11.22	20.4	0.5
Denver	13.83	17.08	0.54	72.34	93.8	0.07	57.8	71.89	0.18
Detroit	19.8	30.41	0.36	25.42	47.38	0.29	36.66	52.67	0.18
Houston	8.8	30.78	0.29	11.4	39.78	0.18	11.82	44.9	0.11
Indianapolis	11.99	14.09	0.68	10.63	11.38	0.61	12.84	14.99	0.57
Los Angeles	18.97	42.17	0.07	26.59	54.23	0.04	26.15	54.11	0.04
Louisville	10.59	16.21	0.57	10.79	16.29	0.61	10.75	15.68	0.61
Memphis	20.26	35.76	0.25	14.9	29.93	0.39	13.73	26.5	0.43
Miami	11	21.13	0.46	17.01	34.17	0.04	18.27	34.01	0.07
New York	3.43	14.21	0.61	5.66	25.32	0.39	1.79	7.73	0.82
Oklahoma	22.32	28.81	0.39	33.3	45.14	0.11	34.27	45.5	0.18
Phoenix	26.42	51.49	0.04	20.12	45.83	0.11	22.38	46.04	0.08
Pittsburgh	21.08	39.06	0.11	10.9	20.19	0.46	14.11	27.3	0.29
Portland	5.42	16.52	0.61	4.46	13.7	0.68	4.86	16.61	0.54
Sacramento	19	51.31	0.25	6.23	19.18	0.54	4.33	19.37	0.5
San Francisco	7.8	40.96	0.18	8.58	43.84	0.29	8.65	44.77	0.25
Seattle	6.73	21.82	0.46	10.99	32.15	0.32	10.62	31.88	0.32
Tampa	5.23	21.65	0.46	5.88	20.36	0.46	7.52	26.29	0.29

### 3.5 SARIMA and GBRT 7-day and 28-day ahead for January, February, and March 2021

**Figure 3:**
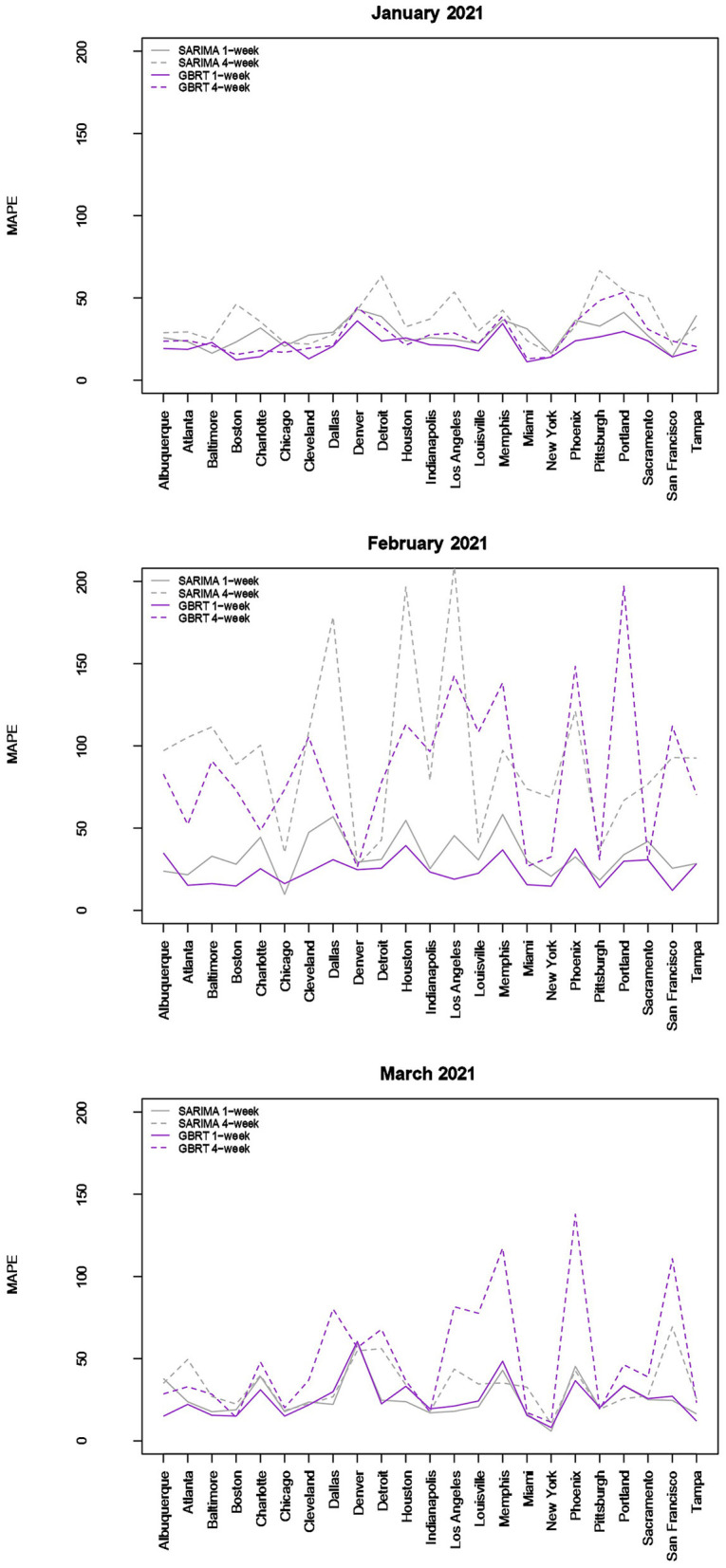
Forecast performance as a function of MSA for January, February, and March 2021. Forecast performance is quantified by MAPE for two different model types and two different forecasting strategies.

A relevant question is whether these findings are robust to the use of other time periods. Figure 3 visualizes the MAPEs associated with 7-day and 28-day ahead SARIMA and GBRT forecasts for the following testing periods: January 1–28 2021; February 1–28 2021; and March 1–28 2021. For the sake of brevity, we only report MAPEs. The proportion of daily forecasts falling within 20% of the actual values for these time periods are available upon request. As discussed, daily case counts dropped significantly for most MSAs during these months, making the evaluation of the predictive abilities an interesting contrast to our previous exercise of constructing forecasts during a period of rising daily COVID-19 cases during November-December 2020. We do not construct forecasts for Oklahoma City and Seattle, given the presence of a significant number of zeros in daily case values that seem anomalous to case counts in other days.

The results visualized in Figure 3 (and tabulated in Tables 5, 6) demonstrate that for almost all MSAs, with respect to 7-day ahead forecasts, GBRTs offer superior predictions relative to SARIMA models for the month of January. GBRT MAPEs are lower than 20% for 9 MSAs, with another 8 MSAs having MAPEs ranging from 20 to 25%. Therefore, for a number of MSAs, GBRTs were able to recognize the change in the trend in daily case counts, from increases to a steady decline. Like the November–December 2020 results, GBRTs are also able to produce more accurate 28-day predictions relative to SARIMA models. GBRT MAPEs are lower than 20% for 6 MSAs, with another 7 MSAs having MAPEs between 20% and 25%. In terms of specific MSAs, Albuquerque, Atlanta, Baltimore, Boston, Cleveland, Louisville, Miami, New York, San Francisco, and Tampa all have GBRT-generated 7-day ahead MAPEs lower than 20% for January 2021, with most of the MSAs (with the addition of Chicago) also possessing low GBRT MAPEs for 28-day ahead forecasts. Including Denver and Indianapolis, these are the same MSAs with low GBRT MAPEs during November - December 2020.

**Table 5:** MAPE values—7-day- and 28-day forecasts from SARIMA models January—March 2021.

**City**	**January 1–28 2021**		**February 1–28 2021**		**March 1–28 2021**	
	**7-day**	**28-day**	**7-day**	**28-day**	**7-day**	**28-day**
Albuquerque	25.78	28.84	24	97.09	37.89	34.88
Atlanta	23.5	29.37	21.82	105.21	23.73	49.47
Baltimore	16.49	24.58	33.04	111.57	17.67	26.88
Boston	23.32	46.21	28.2	88.76	18.85	22.33
Charlotte	31.8	35.54	44.53	100.47	39.12	39.82
Chicago	20.68	22.69	9.91	35.04	17.8	18.46
Cleveland	27.41	21.98	47.47	108.83	23.59	22.42
Dallas	29.13	27.76	57.02	178.42	22.12	26.81
Denver	42.92	42.75	29.45	27.35	59.24	54.72
Detroit	38.63	63.27	31.11	42.85	24.69	56
Houston	24.01	32.47	54.79	196.61	23.84	34.01
Indianapolis	25.9	37.15	25.49	79.09	17.07	17.75
Los Angeles	24.75	53.61	45.55	210.15	17.94	43.55
Louisville	22.38	29.89	30.8	41.36	20.7	34.48
Memphis	36.55	42.52	58.42	97.32	42.85	35.16
Miami	31.29	23.99	30.34	73.81	16.62	32.52
New York	16.44	16.18	20.92	68.7	5.87	10.22
Phoenix	36.39	32.84	32.62	121.21	45.25	42.64
Pittsburgh	32.89	66.6	18.56	37.4	19.95	19.22
Portland	41.2	54.7	33.94	66.91	33.52	25.63
Sacramento	26.99	50.22	42.02	76.86	24.97	27.27
San Francisco	14.49	21.44	25.74	92.98	24.58	69.25
Tampa	39.33	32.47	28.56	92.65	16.36	25.48

**Table 6:** MAPE values—7-day- and 28-day forecasts from GBRT models January—March 2021.

**City**	**January 1–28 2021**		**February 1–28 2021**		**March 1–28 2021**	
	**7-day**	**28-day**	**7-day**	**28-day**	**7-day**	**28-day**
Albuquerque	19.34	23.89	35.19	83.11	14.94	28.43
Atlanta	18.84	24.18	15.52	52.28	22.12	32.84
Baltimore	23.07	21.2	16.49	91.01	15.54	28.31
Boston	12.4	15.76	15.1	73.26	15.01	14.14
Charlotte	14.41	18.21	25.56	48.66	31.05	47.94
Chicago	23.47	17.08	16.49	73.65	15.04	20.08
Cleveland	13.07	19.62	23.55	105.68	21.81	36.84
Dallas	20.8	21.2	31.01	63.77	29.89	80.1
Denver	36.26	44.47	24.97	26.39	60.43	56.56
Detroit	23.86	32.95	25.91	78.08	22.45	67.72
Houston	25.81	21.41	39.58	113.14	33.14	36.39
Indianapolis	21.59	27.82	23.56	96.7	19.48	17.45
Los Angeles	21.12	28.82	19.2	142.85	21.11	81.42
Louisville	17.9	22.39	22.82	108.54	24.27	77.49
Memphis	34.52	39.05	36.97	138.54	48.39	117.08
Miami	11.18	13.1	15.84	26.81	15.43	17.1
New York	14.14	14.26	14.96	32.69	8.03	11.24
Phoenix	24.08	35.68	37.75	148.52	36.63	137.68
Pittsburgh	26.5	48.61	14.07	30.69	20.29	18.92
Portland	29.73	53.59	30.08	197.17	33.57	46.15
Sacramento	23.97	31.12	30.93	30.71	25.67	38.8
San Francisco	14.27	23.95	12.39	112.16	27.09	110.62
Tampa	18.5	20.65	28.56	70.38	12.01	23.09

Some of the same trends are visible for February forecasts with GBRT 7-day ahead MAPEs lower than corresponding SARIMA values for 15 MSAs. Further, 9 MSAs have GBRT MAPEs lower than 20% and 4 MSAs possess GBRT MAPEs for from 20 to 25%. On the other hand, both GBRTs and SARIMA models produce low-quality forecasts with high 28-day ahead MAPEs for the same February time period; 17 or more cities possessing MAPE values >50%, and both methods result in MAPEs of over 100% for 8 MSAs. These results suggest that both GBRT and SARIMA models were unable to accurately predict the downward trend in daily case rates that occurred in February for most MSAs. While we cannot confirm this, a possible reason for the decline in daily case rates might be the increased availability and uptake in vaccinations during and preceding February 2021. To the best of our knowledge, data on city-level vaccination rates are unavailable and, therefore, cannot be included as predictors to test this hypothesis.

In contrast to the February 2021 results, both GBRT and SARIMA March 2021 MAPEs become much lower for 7-day ahead forecasts and are comparable to previous months. GBRT and SARIMA performance are comparable in the sense that the number of MSAs are roughly split between the two methods in terms of which approach has the lowest MAPE. With both methods, 10 MSAs have 7-day ahead MAPEs lower than 20%, while another 8 MSAs possess MAPEs between 20 and 25%. On the other hand, SARIMA seems to be a superior method for generating 28-day ahead predictions in March 2021. Specifically, SARIMA forecasts yield MAPEs that are either comparable or much lower than corresponding MAPE values from GBRT models for most MSAs.

To summarize, the GBRT and SARIMA models are the most reliable in terms of producing 7-day ahead daily predictions with the lowest MAPE values for November-December 2020. With respect to 28-day ahead forecasts for the same time-period, while SARIMA and GBRT MAPES are comparable for several MSAs, there are MSAs where SARIMA MAPEs are much higher. Using Apple Mobility data does not considerably improve forecasts from SARIMA models. GBRT models similarly outperform SARIMA models for 7-day and 28-day ahead predictions for January, but neither model produces high quality predictions for February 2021, with MAPE values from GBRT models, on average, being slightly lower. For March 2021, 7-day ahead forecasts from GBRT and SARIMA models are comparable, with 28-day ahead SARIMA MAPE values being somewhat lower than corresponding GBRT estimates.

## 4 Discussion

Findings from previous studies suggest that the standard SIR model used by epidemiologists for disease predictions resulted in inaccurate forecasts for multiple jurisdictions. Further, we are unaware of any other study that has attempted to construct daily forecasts of a panel of U.S. cities. While there is an abundance of research based on country-level data, research attempting to predict daily COVID-19 cases across cities is scarce (11). This is unfortunate, as cities or MSAs have often been the center of significant occurrences and spread of COVID-19 infections. Besides difficulties associated with obtaining city level data, forecasting daily cases is extremely challenging given the considerable heterogeneity in daily case trends across cities. Perhaps even more important are factors such as differences in testing rates, lack of uniformity in data collection protocols, and inaccurate processing of data, that make comparisons of cases across jurisdictions difficult (30).

This study explores the efficacy of SIR, LME, SARIMA, and GBRT models in producing one and four-week ahead predictions. The choice of these models is premised on different advantages. GBRTs are an example of supervised ML methods that have proved to be effective in different cases (12, 24) and can be developed with less knowledge than more sophisticated machine learning methods, such as deep learning. LME models accommodate city-level heterogeneity, enabling the researcher to borrow information across geographies, and hence do not rely exclusively on time-series variation within geographies. SIR models are the conventional workhorses in epidemiology, but do not have the flexibility to accommodate time-specific changes in external factors, such as population mobility, that can plausibly impact spread of infections. SARIMA models are relatively simple to implement and as noted earlier, are useful when the modeled data have pronounced trend and seasonality. These models have been employed by other studies (11, 12).

We evaluate model performance primarily through MAPEs and find that the prediction accuracy associated with LME and SIR forecasts tends to be inferior relative to that of SARIMA and GBRT forecasts. Compared to SARIMA, GBRTs generate 7-day ahead and 28-day forecasts with lower MAPEs for a vast majority of MSAs for most months. While SARIMA produces lower MAPEs for 28-day ahead predictions for March 2021, the good performance of GBRTs more broadly make it a suitable choice for modeling and forecasting daily COVID-19 case counts during time periods of both increase and decrease in infections. From a policy perspective, these results are important as they imply the availability of superior forecasting methods relative to conventional epidemiological methods. The performance of GBRTs should be noted, given that they are relatively straightforward to use through available statistical packages. The performance of these models is comparable to findings from other studies that use more disaggregated data than at the country level. For example, using provincial level data from Canada, Sen et al. (24) find MAPEs from GBRTs ranging from 8 to 30% for 2 week ahead daily forecasts. Zhang et al. (26) obtain Mean Absolute Percentage Errors ranging from 4 to 8% for 8 counties in California, but not for time-periods of 4 weeks or over.

**Figure 4:**
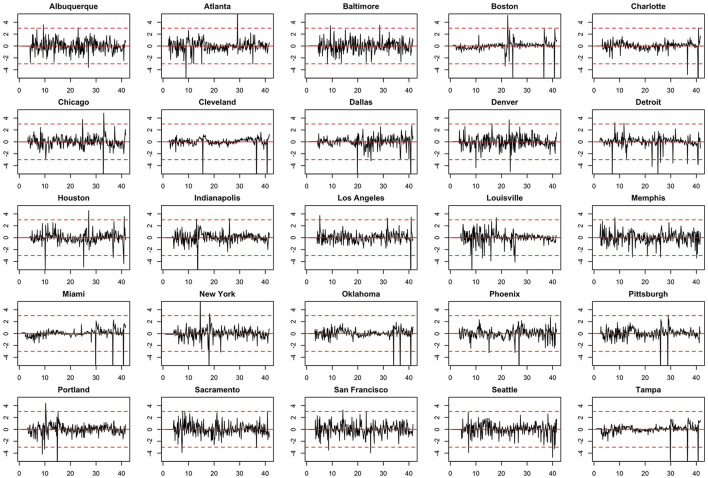
Model residuals associated with the SARIMA model fit to March 2020-December 2020 data.

**Figure 5:**
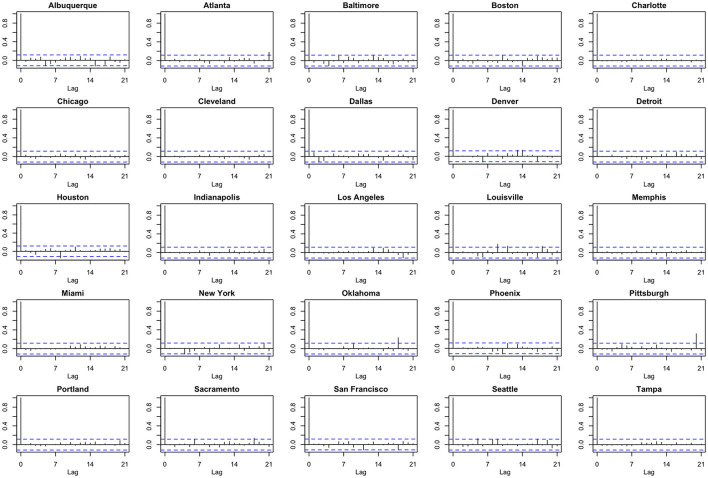
Autocorrelation function (ACF) plots of model residuals associated with the SARIMA model fit to March 2020-December 2020 data.

**Figure 6:**
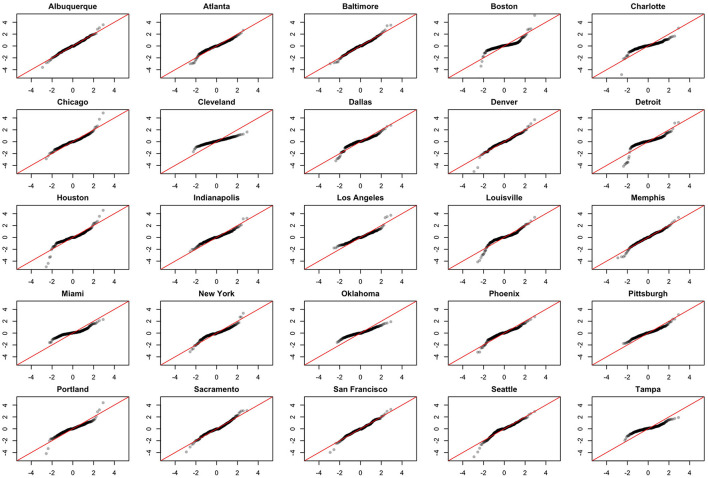
Normal QQ-plots of model residuals associated with the SARIMA model fit to March 2020-December 2020 data.

We note the following limitations. It is important to acknowledge that several of the chosen models make strong and potentially restrictive assumptions including, but not limited to, errors that are independently and identically distributed normal random variables. Such assumptions have been evaluated using a battery of residual diagnostic tools. Although many models and many specifications of these models were considered, we present results only for those whose assumptions were reasonably satisfied. To demonstrate this, we include Figures 4–6 which depict residuals, autocorrelation function and normal quantile-quantile plots of the residuals of the SARIMA model fit to the March 2020-December 2020. As is evident, we do not observe serious violations of stationarity or normality.

## 5 Conclusion

Along with other papers that have emerged over the past 2 years, the results of this study suggest that GBRTs can also be used for predicting the spread of highly infectious diseases on a daily basis. These findings suggest that the relatively basic ML modeling can lead to vital insights for government resource allocation and decision-making, and result in superior disease surveillance relative to conventional epidemiological methods. Future work will investigate the benefits of employing more complex deep learning and neural network-based methods, which have the trade-off of being more complex and possibly more accurate but also more difficult to interpret, especially for a wider audience of policy practitioners.

## Data availability statement

The original contributions presented in the study are publicly available. This data can be found here: https://github.com/kentranz/socialMobilityCOVID/blob/master/data/all.csv.

## Author contributions

AS: Conceptualization, Data curation, Formal analysis, Investigation, Methodology, Project administration, Writing—original draft, Writing—review & editing. NS: Conceptualization, Data curation, Formal analysis, Investigation, Methodology, Software, Validation, Writing—original draft, Writing—review & editing. NT: Data curation, Formal analysis, Investigation, Methodology, Software, Writing—original draft. RA: Data curation, Formal analysis, Investigation, Methodology, Software, Writing—original draft. QZ: Data curation, Formal analysis, Investigation, Methodology, Software, Writing—original draft. JD: Conceptualization, Data curation, Formal analysis, Investigation, Methodology, Software, Supervision, Writing—original draft, Writing—review & editing.

## Footnotes

^1^Please see https://www.cbc.ca/news/health/canada-who-pandemic-no-longer-emergency (last accessed June 1st 2023) for further details.

^2^ https://github.com/nytimes/covid-19-data

^3^ https://coronavirus.jhu.edu/map.html

^4^See, for example, https://www.click2houston.com/news/local/2020/09/23/explained-why-nearly-15000-new-coronavirus-cases-were-reported-in-the-houston-area-in-one-day/ and https://www.wsbtv.com/news/local/georgia-records-highest-daily-increase-coronavirus-cases-since-bef7IAY2EPRURABPICVYA3PO7AEJ4/.

^5^Only the Walking Index was employed in these specifications because of a high correlation with the Driving Index (r = 0.85) and the fact that the Transit Index was consistently insignificant in preliminary regressions that contained all three mobility indicators. More complicated forms of autocorrelation are handled differently in the various models, but generally through the inclusion of lagged case rates as predictor variables.

^6^*B* is a notational operator that shifts subscripts *t* back by 1: *BY*_*t*_ = *Y*_*t*−1_. And when raised to the *k*^th^ power, it shifts subscripts *t* back by *k*: *B*^*k*^*Y*_*t*_ = *Y*_*t*−*k*_.
